# Identification of COVID-19 Waves: Considerations for Research and Policy

**DOI:** 10.3390/ijerph182111058

**Published:** 2021-10-21

**Authors:** Andrés Ayala, Pablo Villalobos Dintrans, Felipe Elorrieta, Claudio Castillo, Claudio Vargas, Matilde Maddaleno

**Affiliations:** 1 Departamento de Matemática y Ciencia de la Computación, Facultad de Ciencias, Universidad de Santiago, Santiago 9170022, Chile; andres.ayala@usach.cl (A.A.); felipe.elorrieta@usach.cl (F.E.); claudio.vargas@usach.cl (C.V.); 2 Programa Centro Salud Pública, Facultad de Ciencias Médicas, Universidad de Santiago, Santiago 9170022, Chile; claudio.castillo.c@usach.cl (C.C.); matilde.maddaleno@usach.cl (M.M.)

**Keywords:** COVID-19, waves, public health, public policy

## Abstract

The identification of COVID-19 waves is a matter of the utmost importance, both for research and decision making. This study uses COVID-19 information from the 52 municipalities of the Metropolitan Region, Chile, and presents a quantitative method—based on weekly accumulated incidence rates—to define COVID-19 waves. We explore three different criteria to define the duration of a wave, and performed a sensitivity analysis using multivariate linear models to show their commonalities and differences. The results show that, compared to a benchmark definition (a 100-day wave), the estimations using longer periods of study are worse in terms of the model’s overall fit (adjusted R^2^). The article shows that defining a COVID-19 wave is not necessarily simple, and has consequences when performing data analysis. The results highlight the need to adopt well-defined and well-justified definitions for COVID-19 waves, since these methodological choices can have an impact in research and policy making.

## 1. Introduction

The COVID-19 pandemic became a major global event since its identification in 2019. During 2020, countries around the world dealt with the crisis under a scenario of uncertainty, including what will happen in 2021 [1,2]. The second year of the pandemic brought new analyses and changes that need to be considered when looking at the impact of COVID-19 in different settings [3,4]. The availability of vaccines, the rise in new variants, and the lessons from the experiences of 2020 pose new challenges for policymakers around the world.

Some of the issues that are relevant to extract lessons and think of solutions are whether there have been several waves of the disease, how to identify them, and if they are different [5,6,7,8]. From a policy perspective, the identification of “waves” is relevant to generate data and analysis for decision making [9].

These differences in the evolution of the pandemic can be found between countries and within geographical regions, where COVID-19 infections can be decomposed into a set of asynchronous sub-trajectories originating from different regions within a country [10].

Despite how popular the concept of “wave” has become, there is no standard definition to identify them. This lack of an operational definition can lead to confusion when studying and communicating about the pandemic. For this, it is important to dedicate efforts to establishing objective methodologies to define COVID-19 waves, allowing both researchers and decision makers to determine and debate about them.

Chile represents an interesting case of study for several reasons. First, it is one of the countries that has been hit more severely by the pandemic [11]. Second, it has been actively proposing new strategies to deal with the pandemic, including the successful mass vaccination process [12,13]. Third, it is a developing, Latin American, unequal country that can be viewed as a reference for the region and other countries. Particularly, it has been shown in previous studies that the pandemic has had an uneven impact within the Metropolitan Region of Chile [14,15]. 

Considering these features, the goal of this study is—using data from the Metropolitan Region, Chile—to propose a particular method to identify COVID-19 waves and show the non-triviality of choosing different measures to define these waves. The results seek to highlight the importance of defining a baseline method with explicit criteria to identify these waves when performing research on COVID-19 in a determined time and location, and for evidence-based decision making. The results can be used to extend the analysis of the ongoing impact of COVID-19 in Chile, and can be applied—mutatis mutandis—in other contexts.

## 2. Methods and Materials

### 2.1. Statistical Strategy

Based on the number of weekly cases—with a PCR-positive test—for each municipality, cumulative incidence rates were calculated as the ratio between the number of cases each week and the estimated population in each municipality, using the projections from the last available census [16] per 100,000 inhabitants. For each municipality, the following thresholds were identified:

- Starting date: week of the first reported case.
- End of first wave (criterion 1): first week (since the starting date) that fulfilled the following conditions:
  1. Weekly incidence rate lower than 70 cases per 100,000 people (70/100,000 cases);
  2. Negative growth incidence rate for at least two consecutive weeks.
- Start of second wave (criterion 2): first week (since the end of the first wave) that fulfilled the following conditions:
  1. Weekly incidence rate higher than 70/100,000 cases;
  2. Positive growth incidence rate in at least one week in which the municipality presented over 70/100,000 cases.
- Average threshold (criterion 3): average week between the end of the first wave and the start of the second wave.

The identification strategy contains the following two criteria: levels and changes. The criterion of 70 cases per 100,000 people was established following the government’s strategy to define geographical COVID-19 measures, the so-called step-by-step plan (Plan Paso a Paso), in which geographically determined restrictions were set according to epidemiological indicators [17]. According to this strategy, a daily incidence rate of 10 cases per 100,000 people was considered as positive and, consequently, it triggered advances in terms of the stringency of measures [18]. In terms of changes, the use of more than one week with low/high cases allows a trend to be identified, avoiding defining a wave based on outliers. Once these thresholds were defined, the duration of waves was calculated as the difference between the starting and end dates for each unit of analysis (municipality), using the three different criteria described above.

To examine the potential effect of the choice between definitions, the cumulative incidence of confirmed cases and deaths due to COVID-19 was calculated for each municipality over the three periods. These first six variables were calculated as the number of COVID-19 confirmed cases/deaths per 100,000 people in each unit of analysis according to the period studied. Based on the above calculation, each of these was divided by the duration of the first wave in days (according to the corresponding criterion), thus obtaining variables adjusted for the duration of each wave.

As an exercise to test the possible effects of the choice between the different definitions, a sensitivity analysis was performed, using the previously described variables as dependent variables in a series of multivariate linear regressions for the 52 municipalities of the Metropolitan Region, Chile. These models were estimated using ordinary least squares (OLS), where the explanatory variables correspond to different demographic, health and socioeconomic factors of each unit of analysis, calculated from information from the CASEN 2017 [19]. Then, for each model, an automatic selection algorithm (stepwise) of explanatory variables was used, simplifying the number of independent variables to the subset that minimizes the AIC criterion. In addition, for the residuals of each estimated model, the Moran’s I test was calculated to detect the existence and degree of spatial autocorrelation.

Finally, the simplified models were compared with the results obtained in the previous study “COVID-19 incidence and mortality in the Metropolitan Region, Chile: Time, space, and structural factors” in order to analyze whether the results vary according to the choice of criteria used to determine the duration of the wave. 

### 2.2. Data

The study uses the number of cases—defined as the sum of confirmed and probable cases—as the main input to identify COVID-19 waves. According to the Chilean Ministry of Health, a confirmed case corresponds to a person who has a positive result of SARS-CoV-2 from an RT-PCR test or who has a positive result from an antigen test for SARS-CoV-2, since it was a suspected case (has symptoms, possible reinfection or has a serious respiratory infection that requires hospitalization) [20,21]. 

The variable was collected weekly, starting on 30 March 2020 to 9 August 2021. For the study, official information coming from the Chilean Ministry of Health (MINSAL) and published by the Ministry of Science was used [22]. The number of deaths due COVID-19 was obtained from open data provided weekly by the Department of Health Statistics and Information (DEIS) [23]. All the data used for this study correspond to data coming from public and open sources, and consider information for the 52 municipalities in the Metropolitan Region, Chile. 

## 3. Results

Table 1 shows the descriptive statistics of the sample, using the different criteria previously described. First, the wave’s duration varies, on average, between 130 and 300 days, using the end of the first wave or the beginning of the second as the criterion. As expected, the cases and deaths follow the same pattern. When adjusting for each municipality’s wave duration, the daily cases range between 15.27 and 25.79, and the daily deaths between 0.59 and 0.95. Finally, the presence of spatial correlation is observed in all the cases.

**Figure 1:**
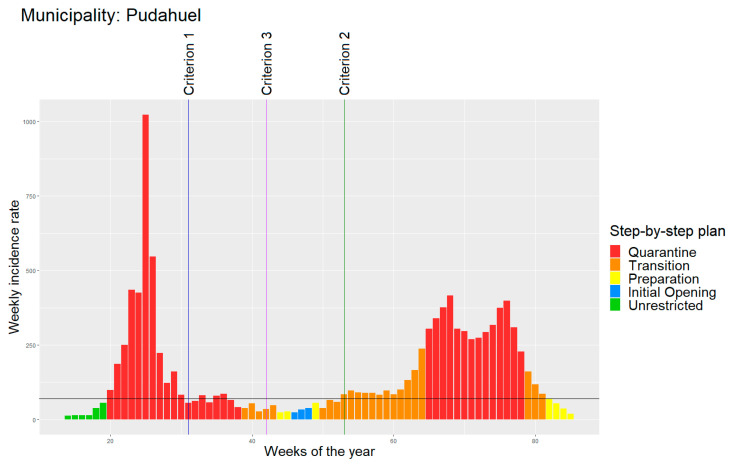
Waves’ identification example: Pudahuel.

Figure 1 shows an example of the identification strategy, using the municipality of Pudahuel (253,139 inhabitants) [16]. First, the x-axis registers the number of cases per 100,000 people in the municipality, while the weeks of the year are on the y-axis. Second, the horizontal line depicts the threshold of the 70/100,000 cases used to identify the start/end of waves. Third, the vertical lines identify the thresholds for the wave’s duration, using the following three previously described criteria: blue = criterion 1; purple = criterion 2; green = criterion 3. Finally, the colors in the bars identify the different stages of the step-by-step strategy, by level of stringency, as follows: red = stage 1 (quarantine); orange = stage 2 (transition); yellow = stage 3 (preparation); blue = stage 4 (initial opening); green = stage 5 (no restriction).

**Figure 2:**
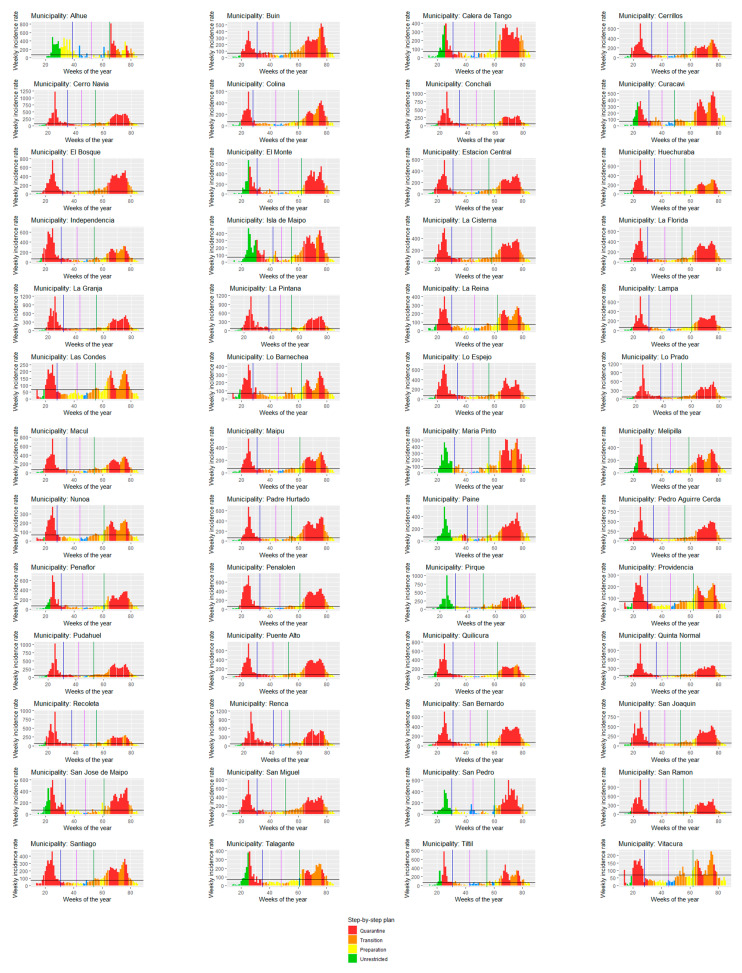
Cases, wave thresholds, and step-by-step stages in the Metropolitan Region.

Figure 2 shows this information for the 52 municipalities of the Metropolitan Region. First, it is interesting to note the different patterns arising from the analysis of different units. In terms of the evolution of cases, there are municipalities that exhibit two clearly defined modes in the distribution of cases (urban municipalities), while, in others, the identification of the end of wave 1 is unclear (cases keep moving up and down), or whether there are more than two waves over the period (rural municipalities). Finally, there are differences in the application of the step-by-step strategy, with municipalities facing early and prolonged quarantines, and others having less stringent measures, both during the first wave (2020) and the second wave (2021).

Finally, Table 2 and Table 3 show the results from the regressions using the different criteria and dependent variables. In both cases, the results are contrasted against the ones reported by using a 100-day period.

Table 2 exhibits the results for the number of cases and the cases adjusted by the duration of the wave. First, compared to the benchmark model (first 100 days in each municipality) variables, multidimensional poverty and the use of public transportation are also significant to explain both case rates, and the cases adjusted by duration. Interestingly, population density does not explain variation in the dependent variable when extending the period of analysis, while the distance to a health center becomes significant. In almost all models, the Moran’s I test for residuals appears as significant, showing the presence of spatial autocorrelation in the regression. In terms of the model as a whole, the adjusted R2 is larger for the benchmark model (100 days), while statistical correlation is not removed from the regression when using wave 1 (definitions using the three proposed criteria) versus the benchmark model. However, when looking at cases adjusted by duration, criterion 3 outperforms the rest.

Table 3 shows the results for death-related variables. For this set of models, the results seem more stable, with the same group of variables appearing as statistically significant in every model. As with the cases, when looking at deaths, the best overall fit corresponds to the model using 100 days; while the model using criterion 3 has a larger adjusted R^2^ for deaths/duration. Spatial correlation of residuals is not present in any model.

## 4. Discussion

### 4.1. Perspectives

The study proposes a method that, based on the weekly cumulative rate of cases, allows waves of COVID-19 to be identified. The method, with its three variants, was used to define the study period when analyzing the impact of COVID-19 (using rates and duration-adjusted rates). The results show that period identification and its impact are far from simple.

The different criteria used to define COVID-19 waves show that the decision is not trivial; both the impact and its determinants can be affected by this methodological decision, confirming the need to build a common definition that will serve as a basis for researchers and decision makers when determining COVID-19 waves. In terms of factors affecting COVID-19 cases and deaths, the proposed models seem to work better—in terms of overall fit—in explaining high-incidence periods (e.g., the 100 days and criterion 1 models); when taking into account duration, a conservative criterion (criterion 3) seems to be a reasonable choice to identify the “true” duration of the waves.

The analysis highlights the importance of considering time as a key factor, both to understand the causes and effects of the pandemic, and to design policies to address it; for example, it is relevant to assess governments’ interventions throughout the pandemic [24]. It also emphasizes the need to acknowledge the researchers’ methodological choices and their consequences in the results [25]. Although the data were restricted to analyzing COVID-19 in a particular setting, the analysis could be used—mutatis mutandis—in other settings and for different health problems. 

We hope that this method will contribute to the creation of a formal and common definition of a “wave”, allowing researchers and authorities to study and communicate about the causes and consequences of the pandemic. The goal of the analysis is not to propose the method, but to highlight the need to have one method when studying and deciding about COVID-19 waves. Other approaches have also been proposed (for example, based on the effective reproduction number, R [26]); the study highlights the need to advance towards adopting an objective measure as a way to enhance academic and policy dialogue.

### 4.2. Limitations

The study has several limitations that need to be considered when interpreting the results. First, the initial motivation for the study was to highlight the need to have a “complete picture” for analyzing COVID-19 data; although the study extends the previous (restricted) definition of a period of 100 days, it needs to acknowledge that COVID-19 is still ongoing, and future data can allow new and different types of results. Second, even though the general method to define waves can be replicated in other contexts, it requires adaptation (e.g., the number of cases to define the threshold). Third, the criteria for defining waves are based exclusively on the total number of confirmed cases. As previously stated, alternatively, the analysis can be performed using different variables, such as deaths or cases by COVID-19 variants, and considering testing in the region of study.

## 5. Conclusions

The information presented adds to the current literature on the causes and impact of COVID-19. It can be useful to monitor data; for example, to identify the rise in future waves in the country. We hope that these results motivate other researchers to assess other ways to solve these problems and contribute to fostering an evidence-based debate on COVID-19.

## Figures and Tables

**Table 1:** Wave 1 statistics using different criteria.

Variable	Criterion 1: End of First Wave				Criterion 2: Start of Second Wave				Criterion 3: Average Threshold			
	Mean	Max	Min	Moran I	Mean	Max	Min	Moran I	Mean	Max	Min	Moran I
Duration (days)	130.981	193	95	0.111 *	300.596	340	249	0.253 ***	215.481	240	186	0.070
Cases	3403.787	6281.746	1406.369	0.381 ***	4536.999	6965.738	2689.269	0.336 ***	3940.650	6471.989	1914.770	0.374 ***
Deaths	125.465	271.645	33.464	0.431 ***	174.446	387.238	41.831	0.392 ***	153.225	336.377	33.464	0.411 ***
Cases/duration	25.789	45.613	13.788	0.399 ***	15.270	24.595	8.704	0.426 ***	18.336	30.461	8.664	0.437 ***
Deaths/duration	0.951	2.245	0.314	0.405 ***	0.586	1.340	0.137	0.445 ***	0.712	1.633	0.167	0.411 ***

Significance level: *** *p* < 0.01, * *p* < 0.1.

**Table 2:** OLS regression under different scenarios: case rate.

Variable	First 100 Days	Criterion 1		Criterion 2		Criterion 3	
	Cases	Cases	Cases/Duration	Cases	Cases/Duration	Cases	Cases/Duration
Population density	4.25 × 10^−5^ *		4.276 × 10^−7^ ***		1.444 × 10^−7^		1.898 × 10^−7^
Multidimensional poverty	5.691 ***	7.573 ***	4.146 × 10^−2^ ***	6.466 ***	0.026 ***	7.381 ***	7.577 × 10^−3^
Distance to health center		2.678 **	1.395 × 10^−2^ *	3.121 ***	0.013	2.624 **	0.033 ***
Use public transportation	4.697 ***	5.775 ***		4.381 **	7.614 × 10^−3^ *	5.415 ***	
Difficult getting healthcare	−31.23						
Years of education							0.019
Constant	33.57	−1804.636	1.816	−530.253	−0.626	−1104.243	−0.668
R-squared	0.568	0.531	0.427	0.489	0.500	0.519	0.507
Adjusted R-squared	0.532	0.502	0.391	0.457	0.458	0.489	0.465
Moran’s I (residuals)	0.081	0.11979 *	0.180 **	0.131 **	0.141	0.143 **	0.168 **

Significance level: *** *p* < 0.01, ** *p* < 0.05, * *p* < 0.1.

**Table 3:** OLS regression under different scenarios: death rate.

Variables	First 100 Days	Criterion 1		Criterion 2		Criterion 3	
	Deaths	Deaths	Deaths/ Duration	Deaths	Deaths/ Duration	Deaths	Deaths/ Duration
People 65+	0.147 *	0.216 × 10^−1^ *	1.783 × 10^−3^ **	0.292 *	9.697 × 10^−4^ *	0.256 *	1.185 × 10^−3^ *
Population density	2.61 × 10^−6^ ***	3.081 × 10^−6^ ***	2.194 × 10^−8^ ***	3.328 × 10^−6^ **	1.206 × 10^−8^ ***	3.143 × 10^−6^ ***	1.504 × 10^−8^ ***
Rurality	−0.056 ***	−0.083 ***	−6.043 × 10^−4^ ***	−0.120 ***	−3.910 × 10^−4^ ***	−0.118 ***	−5.235 × 10^−4^ ***
Multidimensional poverty	0.115 *	0.174 **	8.034 × 10^−4^ *	0.163 *	6.161 × 10^−4^ *	0.182 **	7.561 × 10^−4^ **
Distance to health center	−0.0016						
Cumulative incidence	0.023 ***	0.022 ***	0.023 ***	0.023 ***	0.024 ***	0.020 ***	2.179 × 10^−2^ ***
Constant	−15.59	−23.61	−0.108	−12.04	−0.071	−5.104	−0.039
R-squared	0.805	0.7845	0.7214	0.678	0.7155	0.725	0.726
Adjusted R-squared	0.780	0.7611	0.6911	0.643	0.6845	0.695	0.696
Moran’s I (residuals)	−0.0056	−0.032755	−0.04043	−0.020	−0.013448	−3.154 × 10^−5^	−0.001

Significance level: *** *p* < 0.01, ** *p* < 0.05, * *p* < 0.1.

## Data Availability

Data used in the study is available at: https://github.com/MinCiencia/Datos-COVID19.
